# Human papillomavirus genotypes among women with or without HIV infection: an epidemiological study of Moroccan women from the Souss area

**DOI:** 10.1186/s13027-015-0040-y

**Published:** 2015-12-08

**Authors:** Essaada Belglaiaa, Hicham Elannaz, Bouchra Mouaouya, Mohamed Aksim, Mariette Mercier, Jean-Luc Prétet, Said Chouham, Christiane Mougin

**Affiliations:** Laboratoire de Biologie Cellulaire et Génétique Moléculaire, Faculté des Sciences, Université Ibn Zohr, BP8106, Agadir, 80000 Maroc; EA 3181, Lab Ex LipSTIC ANR-11-LABX-0021, Université de Franche-Comté UBFC, F-25000 Besançon, France; Laboratoire de biologie moléculaire, 5ème Hôpital Militaire, Guelmim, Maroc; Service d’anatomopathologie, Hôpital Hassan II, Agadir, Maroc; Laboratoire de Biologie Cellulaire et Moléculaire, Inserm CIC 1431, CHRU Jean Minjoz, Boulevard Fleming, 25000 Besançon, France

**Keywords:** HPV, HIV, Pap smear, Cervical cancer, Morocco

## Abstract

**Background:**

Data on Human PapillomaVirus (HPV) infection are scarce in Morocco. The objective of the study was to determine the prevalence of HPV and cervical cytology abnormalities in women from the Souss area, Morocco.

**Methods:**

Two hundred and thirty two women who attended the Hassan II hospital (Agadir, Morocco) were recruited in this study. Socio-economic data, sexual activity, reproductive life, history of Pap smear, smoking and HIV status were recorded. Cervical samples were taken using an Ayre spatula. Cytology was reported using the Bethesda system. HPVs were first detected by MY09/11 consensus PCR and then genotyped with INNO-LiPA^®^ assay. Data were analyzed using the logistic regression model.

**Results:**

The median age of women was 42 years (18–76 years). HIV prevalence was 36.2 %. Any HPV type prevalence was 23.7 % in the study population, lower in HIV-negative women (13.3 %) than in HIV-positive women (39.3 %). HPV16 was the most prevalent type (6.5 %), followed by HPV53 and HPV74 (3.4 % each). Most women had normal cervical smears (82 %), the remaining were diagnosed with LGSIL (13 %) and HGSIL (5 %). HPV was detected in 17.4 % of normal smears, 43.4 % of LGSIL and 75 % of HGSIL. HIV status was the most powerful predictor of high risk (hr) and probable hr (phr) HPV infection (odds ratio 4.16, 95 % confidence interval 1.87–9.24, *p* = 0.0005) followed by abnormal cytology (OR 3.98, 95 % CI 1.39–11.40, *p* = 0.01), independently of socio-demographic and behavioral risk factors.

**Conclusions:**

In a Moroccan hospital based-population of the Souss area, HPV infections are frequently detected. In addition, high prevalence of hr and phrHPVs and precancerous lesions among HIV-positive women is likely associated with an increased risk of cervical cancer. This highlights the need for HPV and cervical cancer prevention campaigns in Morocco.

## Background

Human papillomavirus (HPV) infection is the most common sexually transmitted infection (STI) worldwide and the necessary causative agent of precancerous and cancerous lesions of the cervix. The role of high risk HPV (hrHPV) in the development of virtually all cervical cancers (CC) has been well established for more than 20 years [1–3]. CC is the second most common cancer in Moroccan women, after breast cancer. Current estimates indicate that the age-standardized incidence of CC among women in Morocco is 14.3 new cases per 100 000 women/year corresponding to 2258 new cases /year, and the death rate due to this disease is 7 per 100 000 corresponding to 1076 cases/year [4].

To date, more than 200 HPV genotypes have been identified. About 40 of them infect the genital tract, of which 12 were classified as carcinogenic, one as “probably carcinogenic” and 12 as “possibly carcinogenic” to humans [5]. HPV types 16 and 18 are responsible for about 70 % of CC worldwide [6] and 60–70 % of high grade lesions, depending on the geographical area [7]. However, HPV infection of the uterine cervix does not always induce cellular abnormalities [8], indicating that other factors play a role in the development of cervical neoplasia. Human immunodeficiency virus (HIV) infection, another STI with extensive public health impact [9], shares many common behavioural risk factors with HPV infection. HPV and HIV coinfection increases the risk of neoplasia of the lower genital tract [10]. Women with HIV infection commonly harbor a broader range of HPV genotypes, often with multiple concurrent HPV infections, compared to HIV-negative women [11, 12]. HIV influences the natural history of HPV by increasing virulence and the likelihood of persistent infection, and also by hastening the time-course of HPV disease [13, 14].

In late 2013, it was estimated that there were 31 000 persons living with HIV in Morocco, with 50 % of the cases having been registered between 2006–2013. The Souss-Massa-Draa region of Morocco is one of the 5 areas with a high HIV burden accounting for 70 % of notified cases [15]. In the Souss area, women who attend public health-care clinics are most of the time not screened and not treated for HPV-associated lesions. Moreover, there is no epidemiological data available on HPV infection in this region and, as such, the prevalence of HPV infection remains unknown. Therefore, in the present study, we evaluated the prevalence and distribution of HPV genotypes, as well as cervical cytology results, in a hospital based-population of women from the Souss area according to demographic and lifestyle factors and HIV status which may influence HPV infection.

## Patients and methods

### Study population and sample collection

Data on cytology and HPV detection were obtained from 232 women 18 years and older who attended the Hassan II Hospital of Agadir between June 2014 and February 2015. Patients consulted physicians for gynecological care or for different clinical symptoms such as vaginal discharge, vaginal bleeding, vulva itching, lower abdominal and pelvic pain, infertility, menorrhagia, or other symptoms suggestive of gynecological disorders. Women who were virgin, pregnant or had a history of hysterectomy were excluded from the study. More than 80 % of the study population have been diagnosed with or without HIV infection in a free and anonymous screening centre (CIDAG) and HIV status was noted in medical records.

Women received counselling regarding HPV infection and cervical cancer screening. They underwent a pelvic examination and cervical samples were obtained using an extended tip “Ayre” spatula. After the conventional cervical smear was spread onto a glass slide, the tip of the spatula was broken off and placed in a container containing 3 mL of universal transport medium specimen (UTM™) and stored at -20 °C until HPV testing. Furthermore, interviews were conducted to record epidemiological data concerning socio-economic data (level of educational, employment), sexual activity (age at first intercourse, number of sexual partners, contraceptive use and duration), reproductive life (age at first pregnancy, number of births and miscarriages, menopause), past history of Pap smear tests, gynecological lesions and smoking.

Informed consent was obtained prior to enrollment of participants. The study was approved by the institutional board of the Hassan II Hospital of Agadir and conducted in accordance with good clinical practices to benefit the patient.

### Cytology data

The smears were processed for Papanicolaou staining and analyzed according to the Bethesda classification as Negative for Intraepithelial Lesion or Malignancy (NILM) or epithelial cell abnormalities, LGSIL (Low Grade Squamous Intraepithelial Lesion) and HGSIL (High Grade Squamous Intraepithelial Lesion).

### DNA extraction from cervical samples

DNA extraction from cervical smears was performed using QIAamp DNA Mini Kit (Qiagen, Courtaboeuf, France). Aliquots of 3 mL of samples were centrifugated at 4000 rpm for 10 min, the supernatant was removed and the pellet was resuspended in 400 μL TE buffer (10 mM Tris–HCl, 1 mM EDTA, pH8). Then, 400 μL of AL buffer (Qiagen) and 40 μL proteinase K (Qiagen) were added and the mix was digested overnight at 56 °C. All lysates were processed according to the manufacturer’s instructions; DNA was eluted in 80 μL of elution buffer and stored at -20 °C until use.

The quality of extracted DNA was controlled by PCR amplification of the β-globin gene using the primers PC04 (5′-CAA-CTT-CAT-CCA-CGT-TCA-CC-3′) and GH20 (5′-GAA-GAG-CCA-AGG-ACA-GGT-AC-3′), which gives a fragment of 265 bp.

### Human Papillomavirus DNA detection and genotyping

HPV DNA was detected by a standard polymerase chain reaction (PCR) protocol with consensus primer pair MY09/MY11 (5′-CGT-CCM-ARR-GGA-WAC-TGA-TC-3′ and 5′-GCM-CAG-GGW-CAT-AAY-AAT-GG-3′), allowing the production of 450-bp fragments in the HPV L1 open reading frame and the detection of more than 40 low- and high-risk genital HPV types [16]. Identification of HPV genotype(s) in samples positive for HPV DNA by MY09/11 PCR was done using the INNO-LiPA HPV Genotyping Extra® (Fujirebio, Courtaboeuf, France).

HPV genotyping was centrally performed at the Department of Cellular and Molecular Biology at the University Hospital of Besançon (France) according to the manufacturer’s instructions. The genotyping test allows the detection of 28 HPV genotypes: 18 high risk (hr) HPV and probable hr (phr) types (HPV-16, 18, 26, 31, 33, 35, 39, 45, 51, 52, 53, 56, 58, 59, 66, 68, 73 and 82), 7 low-risk (lr) (HPV-6, 11, 40, 43, 44, 54 and 70) and 3 HPV types not classified (HPV-69, 71 and 74). HPV amplimers which did not hybridize to any specific probe were considered as uncharacterized (HPVX) [17].

### Statistical analysis

Quantitative and qualitative variables were described as number and percentages. The distribution of HPV genotypes was summarized using frequency distribution and stratified by cytology results (NILM, LGSIL, and HGSIL). Univariate and multivariate logistic regression models were used to identify risk factors associated successively with HPV infection (any HPV and/or hr and phrHPV) and abnormal cytology. Odds ratios (ORs) and 95 % confidence intervals (CI) were used to quantify the association between risk factors and positivity for any HPV and/or hr and phrHPV genotypes or abnormal cytology. A *p*-value <0.05 was considered statistically significant. Statistical analyses were performed using SAS version 9.4 (SAS Institute Inc., Cary, North California, USA).

## Results

### Patient characteristics

Among the study population, 84/232 (36.2 %) women were diagnosed HIV-positive and 105/232 (45.3 %) HIV-negative. HIV status was unknown for 43/232 (18.5 %) women. Table 1 describes the sociodemographic, behavioral and clinical variables. Median age was 42 years (range 18-76). The majority of women were illiterate (66.8 %) and unemployed (84.1 %). More than half were married (56 %), had their first intercourse at age less than 20 (56.8 %), had their first pregnancy after 18 years of age (58.2 %). About 41 % of women had two or three live births, and 36 % reported one miscarriage or more. The majority (66.4 %) reported that they had only one lifetime sexual partner. A history of contraceptive use was noted in three quarters of women (75.4 %) and 51 % of women said they took oral contraception over a period of more than 5 years. According to their answers, 10.3 % of women were smokers, 13 % had past history of gynecological lesions including anal warts, vulvar condylomas, ovarian cysts, uterine polyps, myomas and fibromas and 18 % were postmenopausal. With regard to the history of cervical cancer screening, 81.5 % of the women had never had a Pap smear.

**Table 1:** Sociodemographic, behavioral, and clinical characteristics of Moroccan women participating in the study (*n* = 232)

Characteristic	n (%)
Age (years)	
<45	149 (64.2)
≥45	83 (35.8)
Educational level	
Illiterate	155 (66.8)
Primary school	38 (16.4)
Secondary and high school	39 (16.8)
Employment	
No	195 (84.0)
Yes	37 (16.0)
Marital status	
Married	130 (56.0)
Single + divorced + widowed	102 (44.0)
HIV status	
Negative	105 (45.3)
Positive	84 (36.2)
Unknown	43 (18.5)
Age at first intercourse (years)	
≥20	100 (43.1)
17–19	66 (28.4)
≤16	66 (28.4)
Age at first pregnancy (years)	
≤ 18	97 (41.8)
> 18	135 (58.2)
Number of live births	
0–1	83 (35.8)
2–3	96 (41.4)
≥4	53 (22.8)
Number of miscarriage	
0	149 (64.2)
≥1	83 (35.8)
Lifetime sexual partners	
Single	154 (66.4)
Multiple	78 (33.6)
History of contraception use	
No	57 (24.6)
Yes	175 (75.4)
Oral contraceptive use	
No	77 (33.2)
Yes (alone or in combination)	155 (66.8)
Intra-uterine device use	
No	221 (95.2)
Yes (alone or in combination)	11 (4.8)
Condom use	
No	192 (82.8)
Yes (alone or in combination)	40 (17.2)
Duration of oral contraceptive use (years)	
< 5	114 (49.1)
≥ 5	118 (50.9)
Smoking exposure	
No	208 (89.7)
Yes	24 (10.3)
Past history of gynecological lesions^a^	
No	202 (87.1)
Yes	30 (12.9)
Menopause	
No	190 (81.9)
Yes	42 (18.1)
Number of Pap smears	
Never	189 (81.5)
≥1	43 (18.5)
Pap smear^b^	
NILM	190 (81.9)
LGSIL	30 (12.9)
HGSIL	12 (5.2)

*Abbreviations*: *HIV* Human Immunodeficiency Virus, *NILM* Negative for Intraepithelial Lesion or Malignancy, *LGSIL* Low-Grade Squamous Intraepithelial Lesion, *HGSIL* High-Grade Squamous Intraepithelial Lesion

^a^Past history of gynecological lesions includes 3 anal warts, 3 vulvar condylomas, 7 uterine polyps, 2 myomas, 3 fibromas and 12 ovarian cysts

^b^Performed at entrance in the study

As regards the results of the Pap smears, 190 (81.9 %, 95 % CI [76.9 – 86.9]) were NILM, 30 (12.9 %, 95 % CI [8.6 – 17.2]) were LGSIL and 12 (5.2 %, 95 % CI [2.3 – 8.1]) were HGSIL. In this series, no cervical cancer was detected.

### HPV detection and genotyping

HPV DNA was detected in 23.7 % (55/232) of the cases but 3 samples (1.3 %) could not be genotyped because they did not match any of the specific probes of the assay (HPVX); thus specific HPV genotypes were available for 52 cases. Eighteen women (34.6 %) were infected by a single HPV genotype and 34 (65.4 %) by multiple HPV genotypes. Among women with multiple genotypes, there were 15 women with dual infection (44.1 %), 9 women with triple infection (26.5 %), 4 women with 4 genotypes (11.7 %) and 6 women with 5 or more genotypes (17.6 %). Multiple infections were only half as frequent in HGSIL (33.3 %, 3/9) as in LGSIL (69.2 %, 9/13) and NILM smears (66.6 %, 22/33).

The distribution of HPV genotypes tested in this study is shown in Table 2. HPV16 was the most prevalent genotype among the study population with 4 mono-infected women and 11 women with mixed infections giving a total prevalence of 6.5 % (15/232). HPV53 and HPV74 were the second most frequent genotypes in the overall population (3.4 %, 8/232 each).

**Table 2:** Distribution of specific Human Papillomavirus (HPV) genotypes in all infections (single or multiple) tested in 55 Moroccan women from the Souss area, Morocco

HPV genotypes	Total n (%)	NILM n (%)	LGSIL n (%)	HGSIL n (%)
hrHPV				
16	15 (6.5)	4 (2.1)	7 (23.3)	4 (33.3)
18	7 (3.0)	2 (1.1)	2 (6.7)	3 (25.0)
31	6 (2.6)	4 (2.1)	2 (6.7)	-
33	6 (2.6)	1 (0.5)	4 (13.3)	1 (8.3)
35	2 (0.9)	2 (1.1)	-	-
39	4 (1.7)	2 (1.1)	2 (6.7)	-
45	2 (0.9)	2 (1.1)	-	-
51	5 (2.2)	5 (2.6)	-	-
52	7 (3.0)	5 (2.6)	2 (6.7)	-
56	6 (2.6)	4 (2.1)	1 (3.3)	1 (8.3)
58	5 (2.2)	4 (2.1)	-	1 (8.3)
68	1 (0.4)	1 (0.5)	-	-
82	3 (1.3)	2 (1.1)	1 (3.3)	-
phr HPV				
26	3 (1.3)	2 (1.1)	-	1 (8.3)
53	8 (3.4)	5 (2.6)	3 (10.0)	-
66	5 (2.2)	4 (2.1)	1 (3.3)	-
lrHPV				
6	5 (2.2)	3 (1.6)	2 (6.7)	-
11	1 (0.4)	-	1 (3.3)	-
44	3 (1.3)	3 (1.6)	-	-
54	7 (3.0)	7 (3.7)	-	-
70	4 (1.7)	2 (1.1)	2 (6.7)	-
Additional HPV				
69/71	5 (2.2)	2 (1.1)	3 (10.0)	-
74	8 (3.4)	7 (3.7)	1 (3.3)	-
HPVX	9 (3.9)	8 (4.2)	-	1 (8.3)

*NILM* Negative for Intraepithelial Lesion or Malignancy, *LGSIL* Low Grade Squamous Intraepithelial Lesion, *HGSIL* High Grade Squamous Intraepithelial Lesion

After stratifying by Pap cytology, HPV was found in 17.4 % (33/190) of women with NILM smear, in 43.3 % (13/30) of LGSIL and in 75 % (9/12) of HGSIL. Twenty two specific genotypes were detected in NILM smears, 15 in LGSIL and only 6 in HGSIL namely HPV16, 18, 26, 33, 56 and 58. The grade of cytological abnormalities was highly associated with hr and phrHPV genotypes (*p* = 0.001 for LGSIL and p < 0.0001 for HGSIL) (Table 3). HPV16 was detected in 2.1 % (4/190) of NILM, 23.3 % (7/30) of LGSIL and 33.3 % (4/12) of HGSIL. HPV18 was the second most prevalent HPV genotype in HGSIL (25 %, 3/12) (Table 2).

**Table 3:** Association between infection by any HPV and by hr and phrHPV types and cytology results among Moroccan women from the Souss area, Morocco

Cytology	n (%)	HPV+ n (%)	OR [95 % CI]	p-value	hr and phr^a^ n (%)	OR [95 % CI]	p-value
NILM	190 (81.9)	33 (17.4)	1.00		27 (14.2)	1.00	
LGSIL	30 (12.9)	13 (43.3)	4.25 [1.76–10.26]	0.001	12 (40.0)	4.02 [1.74–9.28]	0.001
HGSIL	12 (5.2)	9 (75.0)	13.97 [3.24–60.26]	<0.001	9 (75.0)	18.10 [4.60–71.16]	<.0001

^a^hr and phrHPV types: 16, 18, 26, 31, 33, 35, 39, 45, 51, 52, 53, 56, 58, 66, 68 and 82. *NILM*, Negative for Intraepithelial Lesion or Malignancy; *LGSIL*, Low Grade Squamous Intraepithelial Lesion; *HGSIL*, High Grade Squamous Intraepithelial Lesion

**Fig. 1:**
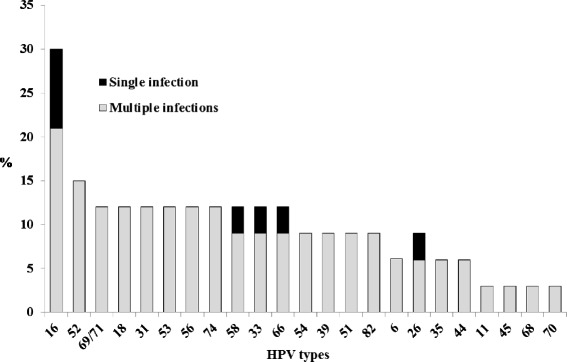
Distribution of HPV genotypes among the 33 HIV+/HPV+ Moroccan women from the Souss area, Morocco. The prevalence of HPV genotypes was calculated by dividing the number of women harboring specific HPV genotype as a single infection (black bars) or multiple infections (grey bars) by the number of HIV+/HPV+ women

After excluding women whose HIV status was unknown, the prevalence of HPV infection was 39.3 % (33/84, 95 % CI 28.8-49.7) in HIV infected women and 13.3 % (14/105, 95 % CI 6.8-19.8) in HIV-negative women. Type-specific HPV distribution in the series of HIV-positive women is shown in Fig. 1; a total of 23 individual HPV types were detected at a frequency of more than 3.0 % in this population. The prevalence of multiple HPV types was higher in HIV-positive women (75.7 % (25/33), 95 % CI 66.5-84.8) compared to HIV-negative women (57.1 % (8/14), 95 % CI 47.6 -66.5). Among the HIV population, HPV DNA was found in 32.2 % (20/62) of NILM smears, 42.8 % (6/14) of LGSIL and 87.5 % (7/8) of HGSIL.

### Risk factors

The association between any HPV, hr and phrHPV, abnormal smear and sociodemographic characteristics as well as sexual behavior was also investigated in the population. By univariate analysis, hr and phrHPV genotypes were associated with number of sexual partners (*p* = 0.04), smoking exposure (*p* = 0.03) and HIV infection (*p* = 0.0004) (Table 4). By multivariate analysis, HIV positivity was associated with a significantly increased risk of hr and phrHPV infection (OR 4.16, 95 % CI 1.87-9.24, *p* = 0.0005) after adjustment for educational level, marital status, age at first pregnancy, number of miscarriages, number of sexual partners and smoking exposure (Table 5).

**Table 4:** Univariate model for identification of factors associated with any HPV, hr and phrHPV genotypes and the presence of abnormal Pap smear

Characteristic	HPV+			hr and phrHPV^a^			Abnormal Pap**		
	n (%)	OR (95 % CI)	*p*-value	n (%)	OR (95 % CI)	*p*-value	n (%)	OR (95 % CI)	*p*-value
Age (years)									
≥ 45	18 (21.7)	1.00		17 (20.5)	1.00		19 (22.9)	1.00	
<45	37 (24.8)	1.19 [0.63–2.26]	0.59	31 (20.8)	1.02 [0.53–1.98]	0.95	23 (15.4)	0.43 [0.22–0.85]	0.01
Educational level									
Secondary and high school	9 (23.1)	1.00		7 (17.9)	1.00		2 (5.1)	1.00	
Primary school	6 (15.8)	0.60 [0.19–1.91]	0.39	4 (10.5)	0.52 [0.14–1.95]	0.33	7 (18.4)	8.35 [0.97–71.65]	0.05
Illiterate	40 (25.8)	1.12 [0.49–2.57]	0.79	37 (23.9)	1.39 [0.57–3.41]	0.47	33 (21.3)	10.00 [1.32–75.68]	0.02
Employment									
Yes	8 (21.6)	1.00		8 (21.6)	1.00		1 (2.7)	1.00	
No	47 (24.1)	1.15 [0.49–2.69]	0.75	40 (20.5)	0.94 [0.40–2.20]	0.88	41 (21)	9.58 [1.27–72.00]	0.03
Marital status									
Married	26 (20.0)	1.00		21 (16.1)	1.00		18 (13.8)	1.00	
Single + divorced + widowed	29 (28.4)	1.59 [0.87–2.92]	0.14	27 (26.5)	1.87 [0.98–3.55]	0.05	24 (23.5)	1.51 [0.77–2.96]	0.22
HIV status									
Negative	14 (13.3)	1.00		13 (12.4)	1.00		12 (11.4)	1.00	
Positive	33 (39.3)	4.21 [2.06–8.58]	<0.0001	29 (34.5)	3.73 [1.79–7.78]	0.0004	22 (26.2)	2.75 [1.27–5.96]	0.01
Age at first intercourse (years)									
≥20	23 (23.0)	1.00		20 (20)	1.00		14 (14)	1.00	
17–19	14 (21.2)	1.15 [0.50–2.64]	0.73	12 (18.2)	1.65 [0.72–3.82]	0.24	12 (18.2)	1.60 [0.63–4.09]	0.32
≤16	18 (27.3)	1.61 [0.73–3.56]	0.24	16 (24.2)	1.15 [0.48–2.77]	0.76	16 (24.2)	2.31 [0.94–5.66]	0.06
Age at first pregnancy (years)									
> 18	28 (20.7)	1.00		24 (17.7)	1.00		22 (16.3)	1.00	
≤ 18	32 (32.9)	1.47 [0.8–2.71]	0.21	24 (24.7)	1.52 [0.80–2.98]	0.20	20 (20.6)	1.19 [0.60–2.32]	0.62
Number of live births									
0–1	22 (26.5)	1.00		18 (21.7)	1.00		14 (63.6)	1.00	
2–3	23 (23.9)	0.87 [0.44–1.72]	0.69	21 (21.9)	1.01 [0.50–2.06]	0.97	21 (21.9)	1.38 [0.65–2.92]	0.40
≥4	10 (18.8)	0.65 [0.28–0.50]	0.30	9 (17)	0.74 [0.30–1.79]	0.50	7 (13.2)	0.75 [0.28–2.00]	0.56
Number of miscarriage									
0	33 (22.1)	1.00		27 (18.1)	1.00		19 (12.7)	1.00	
≥1	22 (26.5)	1.27 [0.68–2.36]	0.45	21 (25.3)	1.53 [0.80–2.92]	0.20	23 (27.7)	2.06 [1.05–4.06]	0.03
Number of sexual partners									
Single	33 (21.4)	1.00		26 (16.8)	1.00		27 (17.5)	1.00	
multiple	22 (28.2)	1.44 [0.77–2.69]	0.25	21 (26.9)	1.93 [1.01–3.70]	0.04	15 (19.2)	0.86 [0.42–1.77]	0.68
History of contraception use									
No	11 (19.3)	1.00		10 (17.5)	1.00		9 (15.8)	1.00	
Yes	44 (25.1)	0.40 [0.67–2.95]	0.37	38 (21.7)	1.30 [0.60–2.82]	0.50	33 (18.8)	1.05 [0.48–2.30]	0.89
Oral contraceptive use									
No	16 (20.7)	1.00		14 (18.2)	1.00		13 (16.8)	1.00	
Yes (alone or in combination)	39 (25.1)	1.28 [0.66–2.48]	0.46	34 (21.9)	1.26 [0.63–2.53]	0.51	29 (18.7)	0.87 [0.43–1.75]	0.70
Condom use									
No	44 (22.9)	1.00		38 (19.8)	1.00	0.46	35 (18.2)	1.00	
Yes (alone or in combination)	11 (27.5)	1.28 [0.59–2.76]	0.54	10 (25)	1.35 [0.61–3.00]		7 (17.5)	1.16 [0.49–2.74]	0.73
Smoking exposure									
No	46 (22.1)	1.00		39 (18.7)	1.00		36 (17.3)	1.00	
Yes	9 (37.5)	2.11 [0.87–5.14]	0.1	9 (37.5)	2.60 [1.06–6.37]	0.03	6 (25)	0.89 [0.29–2.77]	0.84
History of gynecological lesions									
No	49 (24.2)	1.00		43 (21.3)	1.00		36 (17.8)	1.00	
Yes	6 (20.0)	1.28 [0.50–3.31]	0.61	5 (16.7)	1.35 [0.49–3.74]	0.56	6 (20)	1.12 [0.4–3.12]	0.82
Menopause									
No	47 (24.7)	1.00		40 (21)	1.00		33 (17.3)	1.00	
Yes	8 (19.0)	1.40 [0.60–3.23]	0.43	8 (19)	1.13 [0.49–2.64]	0.77	9 (21.4)	0.55 [0.25–1.20]	0.13
Number of Pap smears									
never	48 (25.4)	1.00		41 (21.7)	1.00		37 (19.5)	1.00	
≥1	7 (16.3)	0.57 [0.24–1.37]	0.21	7 (16.3)	0.70 [0.29–1.69]	0.43	5 (11.6)	0.54 [0.20–1.47]	0.22

^a^hr and phrHPV types: 16, 18, 26, 31, 33, 35, 39, 45, 51, 52, 53, 56, 58, 66, 68 and 82. ** *LGSIL*, Low Grade Squamous Intraepithelial Lesion and *HGSIL*, High Grade Squamous Intraepithelial Lesion

**Table 5:** Multivariate analysis^a^ of the characteristics associated with hr and phrHPV types

Characteristics	OR (95 % CI)	*p*-value
Educational level		
Secondary and high school	1.00	
Primary school	0.51 [0.12–2.05]	0.34
Illiterate	1.35 [0.49–3.68]	0.56
Marital status		
Married	1.00	
Single or divorced or widowed	1.17 [0.50–2.73]	0.72
HIV status		
Negative	1.00	
Positive	4.16 [1.87–9.24]	0.0005
Age at first pregnancy (years)		
> 18	1.00	
≤ 18	1.41 [0.66–3.03]	0.38
Number of miscarriages		
0	1.00	
≥1	0.96 [0.45–2.07]	0.92
Number of sexual partners		
Single	1.00	
Multiple	0.71 [0.30–1.70]	0.44
Smoking exposure		
No	1.00	
Yes	2.04 [0.65–6.42]	0.22

^a^The model included all variables with a p-value ≤ 0.2 by univariate analysis

In a second univariate analysis, abnormal Pap smears were associated with unemployment (OR 9.58, 95 % CI 1.27-72.00, *p* = 0.03), number of miscarriages (OR 2.06, 95 % CI 1.05-4.06, *p* = 0.03) and HIV infection (OR 2.75, 95 % CI 1.27-5.96, *p* = 0.01) (Table 4). By multivariate analysis, HIV positivity and number of miscarriages were associated with a significant increase in the risk of abnormal Pap smears (OR 3.98, 95 % CI 1.39-11.40, *p* = 0.01; OR 2.84, 95 % CI 1.14-7.12, *p* = 0.02 respectively) after adjustment for age, educational level, employment, age at first intercourse, menopause and number of Pap smears (Table 6).

**Table 6:** Multivariate analysis*of the characteristics associated with an abnormal cytology (Low Grade and High-Grade Squamous Intraepithelial Lesion)

Characteristics	OR [95 % CI]	*p*-value
Age (years)		
<45	1.00	
≥ 45	0.30 [0.09–1.01]	0.052
Educational level		
Secondary and high school	1.00	
Primary school	3.77 [0.44–32.36]	0.23
Illiterate	3.18 [0.30–33.71]	0.34
Employment		
No	1.00	
Yes	5.86 [0.69–50.13]	0.11
HIV status		
Negative	1.00	
Positive	3.98 [1.39–11.40]	0.01
Age at first intercourse (years)		
≥20	1.00	
17–19	2.41 [0.64–9.08]	0.19
≤16	2.43 [0.69–8.60]	0.17
Number of miscarriages		
0	1.00	
≥1	2.84 [1.14–7.12]	0.02
Menopause		
No	1.00	
Yes	1.33 [0.35–5.12]	0.68
Number of Pap smears		
Never	1.00	
≥1	0.74 [0.20–2.66]	0.64

*The model included all variables with a p-value ≤ 0.2 by univariate analysis as well as variables considered to be important (Pap screening)

## Discussion

In Morocco, cervical cancer (CC) is the second cause of cancer deaths in women after breast cancer, and represents a major public health problem. Here, we observed that more than 80 % of women had never been screened for CC, and the prevalence of HPV infection was high (23.7 %), with differences according to HIV status. Indeed, the rate of HPV was 13.3 % in HIV-negative women and 39.3 % in HIV-positive women. Three Moroccan studies in patients attending the Hospital in Rabat [18, 19] and Casablanca [20] reported results similar to ours. The overall prevalence of HPV was lower (6 %) in the study by Amrani *et al.*, but the number of cytological intraepithelial lesions was low (2.5 % LGSIL and 0.7 % HGSIL) [21] compared to our series (12.9 % of LGSIL and 5.2 % of HGSIL). By contrast, the rate of HPV was high in two geographical areas with high incidence of CC (according to the Rabat and Casablanca Cancer Registries), reaching 32 % in a series of women living in Rabat [22] and 42.5 % in women with normal cytology native of north-central of Morocco [23]. We cannot exclude a bias in the latter studies that did not report HIV status of women. As in most developing countries, some cultural factors can be related to this high HPV prevalence, especially misconceptions and beliefs that prevent people from discussing diseases of the genital tract.

In our study, HPV16 was the most prevalent genotype among the overall HPV-positive population, followed by HPV53 and HPV74. This is in line with studies from around the world, where HPV16 was identified as the most common hrHPV [24–26]. HPV16 and HPV18 represented 3.3 % in NILM smears, 30 % in LGSIL and 58.3 % of HGSIL (Table 2). This finding is consistent with data published recently by the Morocco-ICO Information Centre on HPV and Cancers [4]. Bearing in mind that HPV16 and 18 are responsible for about 70 % of CC cases [3, 27, 28], we suggest the need for HPV screening and anti-HPV vaccination as a primary preventive measure among Moroccan women.

We noted that low risk HPV types were found in 8 % of women with NILM smears and 16.7 % of women with LGSIL but not in women with HGSIL. This confirms that lrHPV rarely cause CIN3 or cancer [29–31].

Interestingly, in the present study, multiple HPV infections were observed in 65.4 % of HPV-positive specimens, but only in 33.3 % of HGSIL cases. Meftah el khair *et al.* reported 34 % of multiple infections in CC from Moroccan women [32]. Co-infection with multiple HPV genotypes has been described in many molecular epidemiologic studies [33–35] and confers an increased risk of high-grade lesions and invasive cervical cancer [36]. This has recently been confirmed in a study including 5,871 sexually active women aged 18-25 years from Costa Rica, in which Chaturvedi *et al.* reported that women with multiple infections were at significantly increased risk of CIN2+ and HGSIL+ as compared with those with single infections [34]. The present findings provide evidence that women with multiple infections should be closely monitored to prevent progression to CC especially in the context of co-infection with HIV.

Reported data on smoking and sexual behavior may be biased because the women may give what is believed to be the desired or socially acceptable answers. This would result in the absence of association between known risk factors with HPV infection in our population.

By contrast, a significant association was found between HIV infection and hr and phrHPV as well as between HIV infection and abnormal cytology. An important clinical consequence of our results is that HIV appears to be the strongest risk factor for HPV infection, independently of the usual socio-demographic and behavioural risk factors.

In the Souss area, the burden of HIV/AIDS is high. In female sex-workers, the prevalence of HIV is above 5 % in Agadir [15]. In recent years, HIV-infected women were referred from the infectious diseases department of the Hassan II Hospital in Agadir to the pathology department of the same Hospital for CC screening at no cost. To the best of our knowledge, there are no previously published studies from Morocco that have investigated the prevalence of HPV genotypes in the population of HIV-positive women, and the relationship between HPV, HIV and cervical cytology. The prevalence of HPV infection in HIV+ women with normal smears (32.2 %) is quite similar to that published in the meta-analysis of Clifford *et al.* reporting a 36.3 % HPV prevalence among 3230 HIV-infected women with no cytological abnormalities [11]. Moreover, multiple infections were found in 75.7 % of HIV-positive women. This is in agreement with the study of Stuardo *et al.* who reported 78.4 % of multiple infections in HIV-infected Spanish women [37] and consistent with data from other studies showing that HIV-infected women not only have a higher prevalence of HPV infection, but are also infected by a wider variety of HPV types than HIV-negative [11, 38, 39]. This has been attributed to lower clearance of HPV [10, 40] or continued sexual exposure to novel HPV types allowing different viral types to settle in the cervical epithelium. Multiple infections could also be due to the reduced systemic and local immunity in HIV-positive women [41].

This study has limitations. The limited number of women included in the study may explain the absence of significant relationship between HPV infection and classical risk factors such as sexual habits. Furthermore, the study population is not representative of the general population of the Souss area. However, this pilot study on a hospital based-population in Agadir is groundwork and a great opportunity to define a more complete research in the future.

In summary, the high prevalence of HPV infection and the diverse distribution of HPV genotypes among Moroccan women from the Souss area emphasizes the need to implement CC screening programs and HPV vaccination for primary prevention [42].
